# Associations Between Two Athlete Monitoring Systems Used to Quantify External Training Loads in Basketball Players

**DOI:** 10.3390/sports8030033

**Published:** 2020-03-11

**Authors:** Aaron Heishman, Keldon Peak, Ryan Miller, Brady Brown, Bryce Daub, Eduardo Freitas, Michael Bemben

**Affiliations:** 1 Department of Athletics, Basketball Strength and Performance, University of Oklahoma, Norman, OK 73019, USA; keldon.peak@ou.edu (K.P.); ryanmiller1@ou.edu (R.M.); brownbrady3@ou.edu (B.B.); eduardofreitas@ou.edu (E.F.); mgbemben@ou.edu (M.B.); 2 Department of Health and Exercise Science, University of Oklahoma, Norman, OK 73019, USA; bdaub34@gmail.com

**Keywords:** athlete monitoring, inertial measurement unit, ultra-wide band, accelerometer, PlayerLoad, ShotTracker, team sport monitoring, off-season training

## Abstract

Monitoring external training load (eTL) has become popular for team sport for managing fatigue, optimizing performance, and guiding return-to-play protocols. During indoor sports, eTL can be measured via inertial measurement units (IMU) or indoor positioning systems (IPS). Though each device provides unique information, the relationships between devices has not been examined. Therefore, the purpose of this study was to assess the association of eTL between an IMU and IPS used to monitor eTL in team sport. Retrospective analyses were performed on 13 elite male National Collegiate Athletic Association (NCAA) Division I basketball players (age: 20.2 ± 1.2 years, height: 201.1 ± 7.6 cm, mass: 96.8 ± 8.8 kg) from three practices during the off-season training phase. A one-way analysis of variance was used to test differences in eTL across practices. Pearson’s correlation examined the association between the Distance traveled during practice captured by IPS compared to PlayerLoad (PL), PlayerLoad per Minute (PL/Min), 2-Dimensional PlayerLoad (PL^2D^), 1-Dimensional PlayerLoad Forward (PL^1D-FWD^), Side (PL^1D-SIDE^), and Up (PL^1D-UP^) captured from the IMU. Regression analyses were performed to predict PL from Distance traveled. The eTL characteristics during Practice 1: PL = 420.4 ± 102.9, PL/min = 5.8 ± 1.4, Distance = 1645.9 ± 377.0 m; Practice 2: PL = 472.8 ± 109.5, PL/min = 5.1 ± 1.2, Distance = 1940.0 ± 436.3 m; Practice 3: PL = 295.1 ± 57.8, PL/min = 5.3 ± 1.0, Distance = 1198.2 ± 219.2 m. Significant (*p* ≤ 0.05) differences were observed in PL, PL^2D^, PL^1D-FWD^, PL^1D-SIDE^, PL^1D-UP^, and Distance across practices. Significant correlations (*p* ≤ 0.001) existed between Distance and PL parameters (Practice 1: r = 0.799–0.891; Practice 2: r = 0.819–0.972; and Practice 3: 0.761–0.891). Predictive models using Distance traveled accounted for 73.5–89.7% of the variance in PL. Significant relationships and predictive capacities exists between systems. Nonetheless, each system also appears to capture unique information that may still be useful to performance practitioners regarding the understanding of eTL.

## 1. Introduction

Collegiate basketball players undergo rigorous training in an attempt to optimize performance. The reactive and intermittent efforts performed during basketball play, including accelerations, decelerations, and frequent change-of-direction movements in all planes of motion, which vary in terms of intensity, distance, duration, and frequency, make understanding the physical demands and stressors an athlete experiences challenging to quantify [1,2]. Wearable technologies have revolutionized the understanding of this movement signature, often referred to as external training load (eTL), or the biomechanical or locomotive stress imposed in the chaotic environment of team sport play, including the volume, direction, and intensity experienced during play [2,3,4,5,6,7]. This eTL during play may be dictated by several factors, including technical/tactical strategies, court size, the number of players in the drill, player experience, player position and more. Nonetheless, it is imperative to use technology that accurately depicts an athlete’s movement characteristics to effectively manage player fatigue, structure training towards optimizing performance, and guide return-to-play protocols following injury [3,8,9].

Outdoor team sports were early adopters of the satellite-based navigational device, global positioning system (GPS), to monitor the position, distance, and speed of player movements, ultimately appraising eTL [6,7]. However, during indoor team sport activities, signal interference eliminates the use of GPS, requiring alternative approaches to assess eTL [10]. Thus, inertial measurement units (IMU), a multisensory device comprised of an accelerometer, a gyroscope, and a magnetometer, is a popular alternative used to characterize athletes’ dynamic movement signature during play in indoor team sports [2,6,7]. Athletes wear the IMU in a supportive harness, specifically designed to unobtrusively secure the unit between the scapulae, in close proximity to their center of gravity, while imposing no interference to the athlete’s movements or play [2]. IMUs offer a portable, practical and convenient solution to monitor eTL [2,5]. Additionally, although IMUs are extensively used in applied practice among collegiate basketball teams, limited data exists characterizing the eTL demands of basketball practice, especially during the off-season training phase, which has no data published to date, but likely exhibits vastly different demands than other phases that parallel the differences in emphasis for physical, technical, and tactical development, as well as the limited time allowed with sport-specific coaches due to league governing body regulations during each phase.

PlayerLoad (PL), expressed as the square root of the sum of the squared instantaneous rate of change in acceleration in each of the three orthogonal planes divided by the scaling factor 100 and expressed in arbitrary units (au), is accepted as a valid and reliable parameter [11,12,13,14], and is frequently reported as the primary workload variable captured by the IMU. Indeed, previous literature has illuminated increases in PL with subsequent change in neuromuscular performance, specifically in basketball athletes [15,16]. Additionally, PL can be broken down into individual vectors (mediolateral, anteroposterior, and vertical), which has been speculated as useful in characterizing the movement signature an athlete experiences [17,18,19]. Interestingly, although the vertical component of PL has been shown to contribute to 50–60% of total PL accumulation during linear running in the laboratory [12] and field-based sports [12,17,20], recent evidence suggests the mediolateral and anteroposterior vectors may contribute to a greater extent during basketball play, reducing the proportional contribution of the vertical PL component to approximately 43% [16]. However, more evidence is needed to solidify these findings observed during basketball play. 

Indoor team sports also utilize indoor positioning systems (IPS), which provide continuous and real-time positional information about a person or an object in an indoor environment [21,22]. Similar GPS technology, IPS are becoming increasingly popular among team sports to track player positioning and their subsequent movements [2,22]. Previously, high costs and the required fixed installation of the system at a venue, limiting mobility of the system, had deterred the use of IPS as a primary tool for monitoring eTL. However, recent advances in technology have led to an increased popularity of IPS used in collegiate basketball, with wide spread adoption across multiple programs and leagues [23,24]. New technology employs ultra-wideband (UWB), a radio frequency signal that disperses information over a wide portion of frequency spectrums, allowing the transmission of large quantities of data with minimal transmit energy, ultimately allowing the detection of player location and movement [21]. The rise in this tracking technology relates to the capability to measure sport-specific indices, such as shooting percentages, shot distribution patterns, assists, and rebounds, among other metrics providing transformative information to the technical and tactical aspects of the game. However, the increase in availability magnifies the ability for the IPS to provide valuable eTL information to performance practitioners and clinicians, such as the total distance an athlete travels during play. Therefore, characterizing the eTL measured by the emerging IPS technology in basketball is warranted.

While both the IMU and IPS provide information regarding eTL, each system may provide unique insights to understanding the associations between the IMU and IPS technologies during basketball play, which may be useful to coaches and practitioners when objectifying and interpreting eTL. Further, establishing the predictive capacity between devices would be useful to practitioners when one system is unavailable or inconvenient to use. Therefore, the present study serves as a preliminary analysis with a two-fold purpose: (1) to characterize the external training loads from both the IMU and the IPS during practices of the off-season training phase in NCAA Division I basketball players; (2) to assess the association between a commercially available IMU and IPS used to monitor eTL in basketball; and (3) to determine the predictive capacity of PL from distance traveled. It was hypothesized there would be a strong association between systems that could generate a significant predictive model to relate the two systems. 

## 2. Materials and Methods

### 2.1. Subjects

A convenience sample of thirteen elite male NCAA Division I basketball players (n: 13, age: 20.2 ± 1.2 years, height: 201.1 ± 7.6 cm, body mass: 96.8 ± 8.8 kg) were included in the present analyses. All subjects were active squad members of the University of Oklahoma’s Men’s basketball team. This research was approved by the Institutional Review Board of the University of Oklahoma.

### 2.2. Study Design

A retrospective analysis was performed to examine the associations between a commercially available IMU (Catapult Innovations, Melborne, VIC, Australia) and commercially available IPS (ShotTracker, Mission, KS, USA) commonly used to monitor eTL in collegiate basketball. Three basketball specific practice sessions were analyzed during the off-season training phase, during which data from both IMU and IPS were simultaneously captured. Collection of the data was then synchronized and exported in order to compare the eTL indices. All practices were sport specific and consisted of both individual and team-based drills, including the majority of time spent in activities that emphasized offensive skill development in both the half and full court setting, but also included a small portion of activities with a defensive emphasis. Both offensive and defensive drills were performed in the half court and full court, and each practice included at least approximately 25% of the time spent in segments of live scrimmage play. The practices were typical of the training phase in collegiate basketball and all practices were designed and implemented by the team’s sport coaches.

### 2.3. Procedures

#### 2.3.1. Inertial Measurement Unit

**Figure 1:**
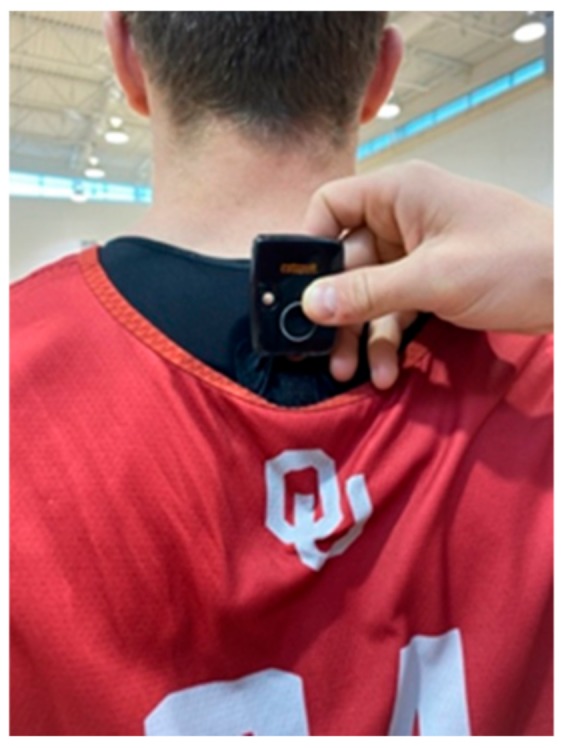
Measurement unit placed in the athlete’s supportive garment.

Participants wore the Catapult Sport OptimEye T6 Unit (Catapult Innovations, Melborne, VIC, Australia) in a supportive harness positioned between the scapulae (Figure 1). All data were collected throughout the duration of practice and ended as the athletes left the floor at the cessation of practice. In parallel with previous work [16], with all participants wearing the same IMU [14] and supportive garment during each practice [18]. All players remained in the respective drill and were not ‘interchanged’ or substituted during practice, even if they were not the primary participant in the drill, as previous literature has suggested that substitutions can artificially inflate training load intensities [10,25]. All data were analyzed via Catapult Sport software (Openfield, Catapult, Innovations, Melborne, VIC, Australia), including the following variables of interest: PlayerLoad™ (PL), PlayerLoad™ per minute (PL/Min), 2-Dimensional PL (PL^2D^), 1-Dimensional PlayerLoad™ Forwards (PL^1D-FWD^), 1-Dimensional PlayerLoad™ Side (PL^1D-SIDE^), 1-Dimensional PlayerLoad™ Up (PL^1D-UP^). PL is a vector of magnitude, expressed as the square root of the sum of the squared instantaneous rate of change in acceleration in each of the three orthogonal planes and divided by the scaling factor of 100 and is expressed in arbitrary units (au), represented mathematically with the following formula:

$$\mathrm{PlayerLoad}™=\sqrt{\frac{{\left(a_{Y1}-a_{Y-1}\right)}^{2}+{\left(a_{X1}-a_{X-1}\right)}^{2}+{\left(a_{Z1}-a_{Z-1}\right)}^{2}}{100}}$$

note: a_Y_ = anteroposterior acceleration; a_X_ = mediolateral acceleration; a_Z_ = vertical acceleration.

Similarly, PL^2D^ only includes triaxial accelerometer data from the mediolateral and anteroposterior planes of movement. PL can be divided into each vector of movement, where 1-Dimensional PlayerLoad™ Forwards (PL^1D-FWD^) assesses movement in the anteroposterior plane, 1-Dimensional PlayerLoad™ Side (PL^1D-SIDE^) assesses movement in the mediolateral plane, and 1-Dimensional PlayerLoad™ Up (PL^1D-UP^) assesses movement in the vertical plane. PL/min divides the PL accumulated by time, providing an intensity index.

#### 2.3.2. Indoor Positioning System

**Figure 2:**
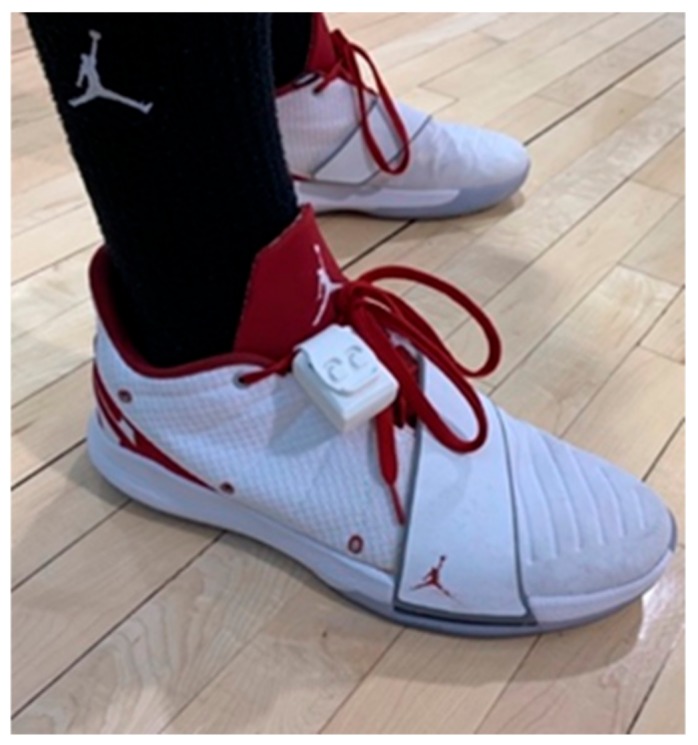
Positioning system (IPS) sensor placed in the protective rubber sheath on the athlete’s shoelace.

Participants wore the IPS sensor (ShotTracker, Mission, Kansas, United States) fastened to the shoe in a protective rubber sheath, looped around the shoelace (Figure 2). In unison with the IMU data collection, monitoring began when the athlete took the court for pre-practice warm-ups, where the sensor on the shoe could be synchronized with the nodes on the ceiling, orienting the unit on the area of play. All data were analyzed and exported using the ShotTracker software (ShotTracker, Mission, Kansas, United States). The variable of interest was Distance traveled (Distance) throughout the duration of practice. 

### 2.4. Statistical Analysis

Data normality was assessed using the Kolmogorov–Smirnov test, as well as skewness and kurtosis. A one-way repeated measure analysis of variance was utilized to examine differences in independent variables across practices. Effects sizes (Cohen’s *d*) were calculated and interpreted as trivial (0–0.19), small (0.20–0.49), medium (0.50–0.79), and large (0.80 and greater) [26]. Pearson’s correlation was used to examine the association between Distance acquired from the IPS and PL, PL/Min, PL^2D^, PL^1D-FWD^, PL^1D-SIDE^, and PL^1D-UP^ captured by the IMU. In accordance with previous literature, correlation coefficients were interpreted as trivial (0–0.09), small (0.10–0.29), moderate (0.30–0.49), large (0.50–0.69), very large (0.70–0.89), and almost perfect (0.90–1) [26]. Additionally, a simple linear regression was performed for each practice to predict PL (PL’) from Distance. Statistical analyses were performed using SPSS, Version 25 (SPSS INC., Chicago, IL). Descriptive statistics are reported as mean ± standard deviation and the level of significance was set at *p* ≤ 0.05. 

## 3. Results

The descriptive statistics for each practice are summarized in Table 1. As additionally outlined in Table 1, there was a significant (*p* < 0.05) difference in PL, PL^2D^, PL^1D-FWD^, PL^1D-SIDE^, PL^1D-UP^, as well as Distance among the three practices observed in this study. There was only a significant difference in PL/min between Practice 1 and Practice 2 (*p* < 0.05). On average across all three practices, PL^1D-FWD^, PL^1D-SIDE^, and PL^1D-UP^ accounted for 28.2 ± 2.5%, 28.9 ± 1.2%, and 42.8 ± 2.2% of total PL accumulation, respectively.

**Figure 3:**
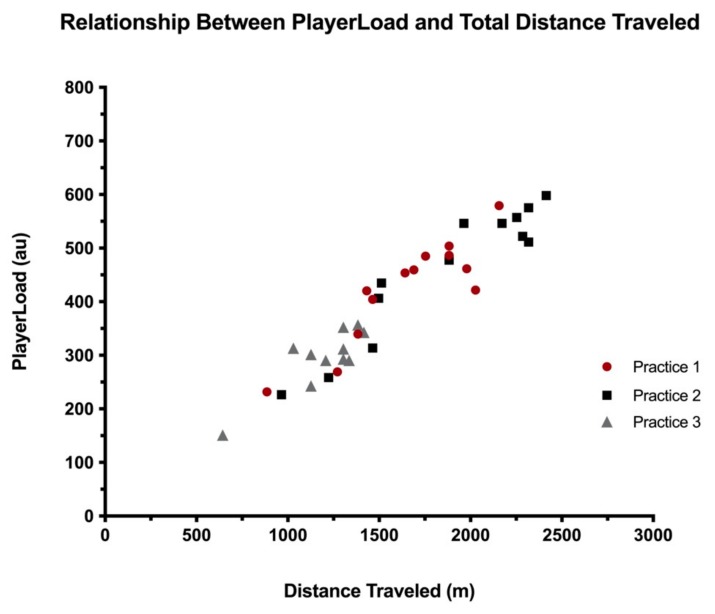
Relationship between PlayerLoad and Total Distance Traveled.

The results of the Pearson Correlations between IPS Distance and the IMU variables of PL, PL/Min, PL^2D^, PL^1D-FWD^, PL^1D-SIDE^, and PL^1D-UP^ are outlined in Table 2. The relationship between PL and Distance for each practice is visualized in Figure 3. Regression analyses offered significant models for each practice, accounting for 79.4% of the variance in Practice 1 (adjusted R^2^ = 0.775; standard error of the estimate (SEE) = 45.4), 89.7% in Practice 2 (adjusted R^2^ = 0.887; SEE = 45.4), and 73.5% in Practice 3 (adjusted R^2^ = 0.706; SEE = 41.5), with each model displaying a very large effect size (Practice 1, f^2^ = 42.4; Practice 2, f^2^ = 95.5; Practice 3, f^2^ = 25.0). The equations to predict PL (PL’) for each practice from Distance (x) were as follows: Practice 1, PL’ = 0.241x + 26.96; Practice 2, PL’ = 0.243x + 5.34; and Practice 3, PL’ = 0.227x + 23.64. 

## 4. Discussion

The purpose of the present study was to characterize the eTL demands of collegiate basketball practice during the off-season training phase, as well as to assess the associations between a commercially available IMU and IPS used to monitor eTL in basketball. The main findings of this study were 1) the unique characterization of eTL demands during practices of the off-season training phase in a cohort of NCAA Division I basketball players, including the novel parameter of Distance traveled; 2) the identification of a large positive correlations between the variables from the IMU and IPS used to capture eTL; and 3) the development of a predictive model to capture the association between Distance traveled and PL over three practices of significantly different eTL.

The present study corroborates previous findings from our research group [16], indicating that the vertical component of PL (PL^1D-UP^) contributes less, while the combined anteroposterior (PL^1D-FWD^) and mediolateral (PL^1D-SIDE^) components contribute to a greater proportion of total PL during basketball play, as compared to traditional linear running. Specifically, laboratory research [12] and outdoor field sports [17] have documented the vertical component contributing approximately 55% to PL, however, this value is reduced to 42.8 ± 2.2% during basketball play in the present study, similar to recent observations [16]. These results are likely related to the large lateral component of basketball, as well as the frequent accelerations and decelerations, dictated by the size of the player and the intermittent nature of the sport. Acceleratory and decelerator movements would produce more horizontal and less vertical ground reaction forces than the top-end speed running [27] commonly performed in the larger areas of play among outdoor sports [17,28]. As previously proposed, these data suggest the vertical component of basketball activity plays a smaller role in masking a minor increase in anteroposterior and mediolateral vectors of movement than that experienced in other sports [16,18,19].

There was a significant difference in eTL variables across the three practices examined in the present study, suggesting a degree of variability in practice workloads during the off-season training phase. While this preliminary analysis provides a glimpse of eTL experienced in the off-season training block, future research is warranted to bolster this understanding. Further, considering the limited contact hours allowed between sport-specific coaches and players during the off-season training block, per NCAA regulations, eTL information may provide a critical link to ensure the augmentation of training adaptations during off-season training programs so athletes are prepared for the rigorous forthcoming seasonal demands. 

The current investigation reported lower PL than recent work during the preseason phase in men’s collegiate players [16]. Similarly, Fox et al. (2018) reported lower PL in a cohort of semi-professional players during sport-specific training and conditioning. In contrast, the present data were higher than that previously reported in collegiate men’s basketball players during the preseason [15,29], as well as higher than that detected in men’s professional players [30]. However, the present study paralleled the PL experienced in both elite NCAA Division I collegiate women’s players during game play [31] and semi-professional men’s players during competition [10]. Numerous factors can influence the eTL experienced during basketball practices and games, which would explain the array of eTL observed during basketball activity currently in the literature, including the training phase, the team’s style of play, player’s skillset, player experience, the number of players in each drill, the size of the playing area, and even the technical or tactical emphasis of a drill. Importantly, to the authors’ knowledge, there are no data published to date outlining the movement demands of men’s collegiate basketball players during competition, which could be critical to sport coaches, as well as strength and conditioning practitioners, in developing a comprehensive annual training plan, including tactical periodization towards optimizing prescribed workloads for maximizing physical preparation.

The average PL/min across practices in the present study were similar to the highest PL/min values previously reported during the preseason in men’s collegiate players [16]. Interestingly, the elevated PL/min values coupled with the lower total PL values in the present study suggests that the primary driver attenuating the total PL observed during this off-season training phase likely relates to the shorter duration of practice in the off-season rather than a moderation in practice intensity. The observed PL/min of the present study was lower than that experienced in elite collegiate women’s players, as well as in professional players [31,32], but greater than semi-professional players during competition [10]. The higher PL/min during the off-season training block, compared to that previously reported during the preseason in collegiate men [16] likely reflects the off-season training block practices incorporating more individual skill development work, with the structure of drills during practice including more players at a time, ultimately reducing the amount of time players are not incorporated in activity. While a variety of factors can influence game intensity, other observed differences with the previous literature likely relate to style and level of play, and potential differences between game and practice intensities. Importantly, not interchanging, or “benching” athletes when they are not the primary participant in an activity, as suggested by previous literature [10,25], likely plays a role in both attenuating the intensity (PL/min) and augmenting the total volume (PL) of the work observed in the present study, and may also explain discrepancies among intensities found in the literature. 

Similar to the differences observed in PL from the IMU, the present study also observed shorter Distance traveled compared to previous research. Previous literature has measured [33,34,35,36] or estimated [10] the total Distance traveled during basketball activities, ranging from 5000–7000 m, which is much higher than the total Distance traveled per practice in the present study. Interestingly, Fox et al. (2018) reported an estimated equivalent distance, which was derived from accelerometer data surpassing 5000 m with an associated PL of approximately 600 (au) on average for a practice, both of which are proportionately greater than the eTL observed in the present study. Again, these differences likely relate to the training phase and drills included in the off-season training block. While not specifically characterized in the present study, the majority of drill work was performed in the half court setting, with focus on individual skill development and less live or simulated game action in the full court. Evidence from alternative sports suggests that the training phase [37] and the number of days out from a competition [28] influence eTL due to tactical periodization strategies. Therefore, these preliminary observations of eTL among collegiate basketball players in the off-season highlights the need for future investigations in this area, which may provide insights useful for enhancements in tactical periodization to ensure coaches and performance practitioners are preparing athletes for the volume of work experienced during the competitive phase of the training cycle. 

There were very large to nearly perfect correlations between Distance and all PL parameters. While the strong correlation between PL and Distance has been documented, to our knowledge this is the first study to identify the significant association among basketball players, albeit a smaller correlation than observed in alternative sports [12,20,38]. Although the parameters exhibit a strong association this does not mean the tools or parameters are interchangeable, and each may provide unique insights. For example, Distance and PL shared 85–95% variance across practices, leaving 5-15% difference that may influence the precision and characterization of an athlete’s movement profile. Although, it may be reasoned that Distance and PL^2D^ should display the highest association since both the IPS Distance and PL^2D^ only measure the anteroposterior and mediolateral planes of motion (both 2-dimensional measurements), these parameters only shared approximately 65–81% variance between measurements across the three practices. Such strong correlations between variables, such as between Distance and PL^1D-UP^, which may appear seemingly less related, likely exist due to a strong collinearity among PL parameters. Interestingly, Practice 2 had the highest training loads overall, while also exhibiting the highest correlation among variables. While more observations beyond this preliminary analysis are necessary, these data suggest increases in eTL may result in an improved relationship between monitoring systems. Future research should explore the sensitivity and specificity of each system to characterize the volume of work performed or even examine the combined utility of using information from both systems to maximize the understanding of movement during play, as it relates to internal load responses, as well as subsequent indices of fatigue and recovery.

The present study has limitations that warrant discussion. A limited number of practices were included in the analyses; however, this study serves as a preliminary analysis for future research to couple eTL devices, as well as illuminate typical player eTL profiles experienced during the off-season training block. In addition, these observations may not be generalizable to female collegiate players during the off-season, as previous evidence has suggested female players may cover less distance and have lower movement frequencies than their male counterparts [39]. Furthermore, although the IMU used in the present study has been established as valid and reliable [11,12,14], future investigations should provide more evidence beyond that of the manufacturer solidifying the validity and reliability of the IPS used in the present study.

While not examined in the present study, conflicting data exist ascertaining positional differences in eTL among basketball players [16,31,40,41]. To the author’s knowledge, no available data have examined differences in distance traveled between positions in collegiate basketball athletes, which may be a key index warranting investigation in the future. Additionally, expanding the information available from the IPS to include velocity information, similar to outdoor GPS systems, may be clinically relevant as work rate by velocity zones has been used as key performance indicators among outdoor sports [5,6,28], which may ultimately pair well the sensitivity of the IMU to detect micro-movements and develop the most comprehensive movement signature.

## 5. Conclusions

In conclusion, this study outlined the eTL characteristics experienced during the off-season training block from two different athlete monitoring systems. Additionally, the present study offers an ecologically valid analysis of the association between the IMU and IPS technologies commonly used in the collegiate basketball setting, which demonstrated a strong correlation. Further, although each device provides unique information regarding each individual athlete, we have identified significant relationships, and thus a high predictive capacity between devices, which may be useful for practitioners when one device is not available. These monitoring devices captured significantly associated information, which creates a predictive capacity between devices. Nonetheless, each device also appears to capture unique information, which may be useful in developing individual player profiles and maximizing the understanding of eTL during play. The present study offers key practical applications for basketball performance practitioners. Firstly, this study offers insight into the eTL experienced by Division I NCAA men’s collegiate basketball players during sport-specific, team practices in the off-season training phase. Additionally, this preliminary study establishes the relationship between the LPS and IMU data collected during basketball play, which can be useful to researchers when comparing eTL measured with different devices in the literature, as well as useful to practitioners in estimating eTL when either the LPS or IMU device is unavailable.

## Figures and Tables

**Table 1:** Descriptive statistics of external training load variables during each practice.

Variable	Practice 1	Practice 2	Practice 3	Effect (*d*) Practice 1–2	Effect (*d*) Practice 2–3	Effect (*d*) Practice 1–3
PL (au) *##††	420.4 ± 102.9	472.8 ± 109.5	295.1 ± 57.8	0.49	2.03	1.50
PL/Min (au/min) *	5.8 ± 1.4	5.1 ± 1.2	5.3 ± 1.0	0.54	0.18	0.41
PL^2D^ (au) *##††	279.4 ± 70.2	311.4 ± 73.2	194.9 ± 42.5	0.45	1.95	1.46
PL^1D-UP^ (au) *##††	265.9 ± 65.7	302.1 ± 70.4	187.3 ± 34.4	0.53	2.07	1.50
PL^1D-SIDE^ (au) *##††	180.7 ± 45.4	201.5 ± 49.4	125.3 ± 25.7	0.44	1.94	1.50
PL^1D-FWD^ (au) *##††	175.1 ± 46.9	194.9 ± 46.1	122.78 ± 30.1	0.43	1.85	1.33
IPS Distance (m) *##††	1645.9 ± 377.0	1940.0 ± 436.3	1198.2 ± 219.2	0.72	2.15	1.45

PL = PlayerLoad™; PL^2D^ = 2-Demensional PlayerLoad™; PL^1D-FWD^ = 1-Demensional PlayerLoad™ Forwards; PL^1D-SIDE^ (au) = 1-Demensional PlayerLoad™ Side; PL^1D-UP^ = 1-Demensional PlayerLoad™ Up; IPS = Indoor Positioning System; au = arbitrary units; cts = counts; * = Significant difference between Practice 1 and Practice 2, *p* ≤ 0.05; ## = Significant difference between Practice 1 and Practice 3, *p* ≤ 0.001; †† = Significant difference between Practice 2 and Practice 3, *p* ≤ 0.001. *d* = Effects size (Cohen’s *d*). interpreted as trivial (0–0.19), small (0.20–0.49), medium (0.50–0.79), and large (0.80 and greater) [26].

**Table 2:** Correlation between inertial measurement unit variables and the indoor positioning distance.

	Practice	PL	PL/Min	PL^2D^	PL^1D-FWD^	PL^1D-SIDE^	PL^1D-UP^
IPS (Distance)	Practice 1	0.891 **	0.891 **	0.863 **	0.799 **	0.879 **	0.887 **
	Practice 2	0.947 **	0.947 **	0.901 **	0.819 **	0.944 **	0.972 **
	Practice 3	0.858 **	0.872 **	0.809 **	0.810 **	0.761 **	0.891 **

IPS = indoor positioning system; PL = PlayerLoad™; PL^2D^ = 2-Demensional PlayerLoad™; PL^1D-FWD^ = 1-Demensional PlayerLoad™ Forwards; PL^1D-SIDE^ = 1-Demensional PlayerLoad™ Side; PL^1D-UP^ = 1-Demensional PlayerLoad™ Up; ** = *p* ≤ 0.001.

## References

[1] Stojanović E., Stojiljković N., Scanlan A.T., Dalbo V.J., Berkelmans D.M., Milanović Z. (2018). The Activity Demands and Physiological Responses Encountered During Basketball Match-Play: A Systematic Review. Sports Med..

[2] Fox J.L., Scanlan A.T., Stanton R. (2017). A Review of Player Monitoring Approaches in Basketball: Current Trends and Future Directions. J. Strength Cond. Res..

[3] Halson S.L. (2014). Monitoring Training Load to Understand Fatigue in Athletes. Sports Med..

[4] Bourdon P.C., Cardinale M., Murray A., Gastin P., Kellmann M., Varley M.C., Gabbett T.J., Coutts A.J., Burgess D.J., Gregson W. (2017). Monitoring Athlete Training Loads: Consensus Statement. Int. J. Sports Physiol. Perform..

[5] Dellaserra C.L., Gao Y., Ransdell L. (2014). Use of integrated technology in team sports: A review of opportunities, challenges, and future directions for athletes. J. Strength Cond. Res..

[6] Cummins C., Orr R., O’Connor H., West C. (2013). Global positioning systems (GPS) and microtechnology sensors in team sports: A systematic review. Sports Med..

[7] Chambers R., Gabbett T.J., Cole M.H., Beard A. (2015). The Use of Wearable Microsensors to Quantify Sport-Specific Movements. Sports Med..

[8] Dunlop G., Ardern C.L., Andersen T.E., Lewin C., Dupont G., Ashworth B., O’Driscoll G., Rolls A., Brown S., McCall A. (2019). Return-to-Play Practices Following Hamstring Injury: A Worldwide Survey of 131 Premier League Football Teams. Sports Med..

[9] Taberner M., Allen T., Cohen D.D. (2019). Progressing rehabilitation after injury: Consider the “control-chaos continuum”. Br. J. Sports Med..

[10] Fox J.L., Stanton R., Scanlan A.T. (2018). A Comparison of Training and Competition Demands in Semiprofessional Male Basketball Players. Res. Q. Exerc. Sport.

[11] Boyd L.J., Ball K., Aughey R.J. (2011). The reliability of MinimaxX accelerometers for measuring physical activity in Australian football. Int. J. Sports Physiol. Perform..

[12] Barrett S., Midgley A., Lovell R. (2014). PlayerLoad™: Reliability, convergent validity, and influence of unit position during treadmill running. Int. J. Sports Physiol. Perform..

[13] Barrett S., Midgley A.W., Towlson C., Garrett A., Portas M., Lovell R. (2016). Within-Match PlayerLoad™ Patterns During a Simulated Soccer Match: Potential Implications for Unit Positioning and Fatigue Management. Int. J. Sports Physiol. Perform..

[14] Nicolella D.P., Torres-Ronda L., Saylor K.J., Schelling X. (2018). Validity and reliability of an accelerometer-based player tracking device. PLoS ONE.

[15] Heishman A.D., Curtis M.A., Saliba E., Hornett R.J., Malin S.K., Weltman A.L. (2018). Noninvasive Assessment of Internal and External Player Load: Implications for Optimizing Athletic Performance. J. Strength Cond. Res..

[16] Heishman A.D., Daub B.D., Miller R.M., Freitas E.D.S., Bemben M.G. (2020). Monitoring External Training Loads and Neuromuscular Performance for Division I Basketball Players over the Preseason. J. Sports Sci. Med..

[17] Cormack S., Mooney M., Morgan W., McGuigan M. (2013). Influence of neuromuscular fatigue on accelerometer load in elite Australian football players. Int. J. Sports Physiol. Perform..

[18] McLean B.D., Cummins C., Conlan G., Duthie G., Coutts A.J. (2018). The Fit Matters: Influence of Accelerometer Fitting and Training Drill Demands on Load Measures in Rugby League Players. Int. J. Sports Physiol. Perform..

[19] Davies M.J., Young W., Farrow D., Bahnert A. (2013). Comparison of Small-sided Games on Agility Demands in Elite Australian Football. Int. J. Sports Physiol. Perform..

[20] Polgaze T., Dawson B., Hiscock D., Peeling P. (2015). A comparative analysis of acceleromter and time motion data in elite mens hockey training and competition. Int. J. Sports Physiol. Perform..

[21] Alarifi A., Al-Salman A., Alsaleh M., Alnafessah A., Al-Hadhrami S., Al-Ammar M.A., Al-Khalifa H.S. (2016). Ultra Wideband Indoor Positioning Technologies: Analysis and Recent Advances. Sensors.

[22] Serpiello F.R., Hopkins W.G., Barnes S., Tavrou J., Duthie G.M., Aughey R.J., Ball K. (2018). Validity of an ultra-wideband local positioning system to measure locomotion in indoor sports. J. Sports Sci..

[23] Felts T. (2019). ShotTracker partners with entire NCAA conference, taking shot at potential in-game analytics. Startland News.

[24] Miller K. The Analytics Uprising Is Upon College Basketball: How It Could Alter Sport. https://bleacherreport.com/articles/2807432-the-analytics-uprising-is-upon-college-basketball-how-it-could-alter-the-sport.

[25] Narazaki K., Berg K., Stergiou N., Chen B. (2009). Physiological demands of competitive basketball. Scand. J. Med. Sci. Sports.

[26] Cohen J. (1992). A power primer. Psychol. Bull..

[27] Nagahara R., Mizutani M., Matsuo A., Kanehisa H., Fukunaga T. (2018). Association of Sprint Performance With Ground Reaction Forces During Acceleration and Maximal Speed Phases in a Single Sprint. J. Appl. Biomech..

[28] Ward P.A., Ramsden S., Coutts A.J., Hulton A.T., Drust B. (2018). Positional Differences in Running and Nonrunning Activities During Elite American Football Training. J. Strength Cond. Res..

[29] Heishman A.D., Curtis M.A., Saliba E.N., Hornett R.J., Malin S.K., Weltman A.L. (2017). Comparing Performance During Morning vs. Afternoon Training Sessions in Intercollegiate Basketball Players. J. Strength Cond. Res..

[30] Svilar L., Castellano J., Jukic I. (2018). Load Monitoring System in Top-Level Basketball Team: Relationship Between External and Internal Training Load. Kinesiology.

[31] Ransdell L.B., Murray T., Gao Y., Jones P., Bycura D. (2019). A 4-Year Profile of Game Demands in Elite Women’s Division I College Basketball. J. Strength Cond. Res..

[32] Svilar L., Castellano J., Jukic I. (2019). Comparison of 5vs5 Training Games and Match-Play Using Microsensor Technology in Elite Basketball. J. Strength Cond. Res..

[33] Scanlan A.T., Dascombe B.J., Reaburn P., Dalbo V.J. (2012). The physiological and activity demands experienced by Australian female basketball players during competition. J. Sci. Med. Sport.

[34] Scanlan A., Dascombe B., Reaburn P. (2011). A comparison of the activity demands of elite and sub-elite Australian men’s basketball competition. J. Sports Sci..

[35] Ben Abdelkrim N., El Fazaa S., El Ati J. (2007). Time-motion analysis and physiological data of elite under-19-year-old basketball players during competition. Br. J. Sports Med..

[36] Ben Abdelkrim N., Castagna C., Jabri I., Battikh T., El Fazaa S., El Ati J. (2010). Activity profile and physiological requirements of junior elite basketball players in relation to aerobic-anaerobic fitness. J. Strength Cond. Res..

[37] Jeong T.-S., Reilly T., Morton J., Bae S.-W., Drust B. (2011). Quantification of the physiological loading of one week of “pre-season” and one week of “in-season” training in professional soccer players. J. Sports Sci..

[38] Gallo T., Cormack S., Gabbett T., Williams M., Lorenzen C. (2015). Characteristics impacting on session rating of perceived exertion training load in Australian footballers. J. Sports Sci..

[39] Oba W., Okuda T. (2008). A Cross-sectional Comparative Study of Movement Distances and Speed of the Players and a Ball in Basketball Game. Int. J. Sport Heal. Sci..

[40] Svilar L., Castellano J., Jukic I., Casamichana D. (2018). Positional Differences in Elite Basketball: Selecting Appropriate Training-Load Measures. Int. J. Sports Physiol. Perform..

[41] Schelling X., Torres L. (2016). Accelerometer load profiles for basketball-specific drills in elite players. J. Sports Sci. Med..

